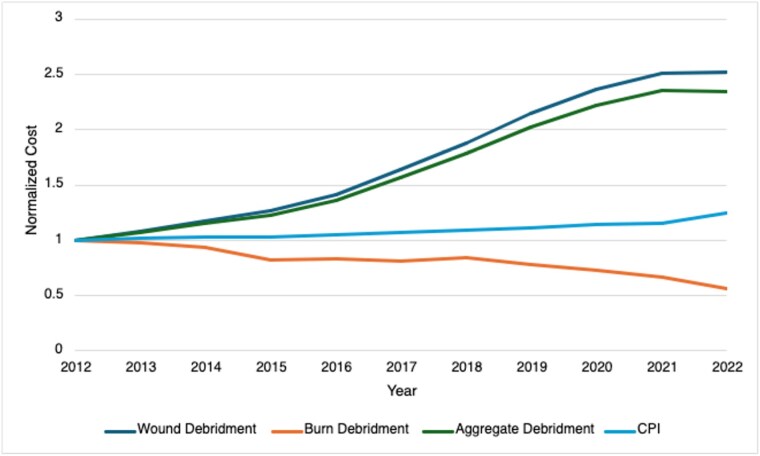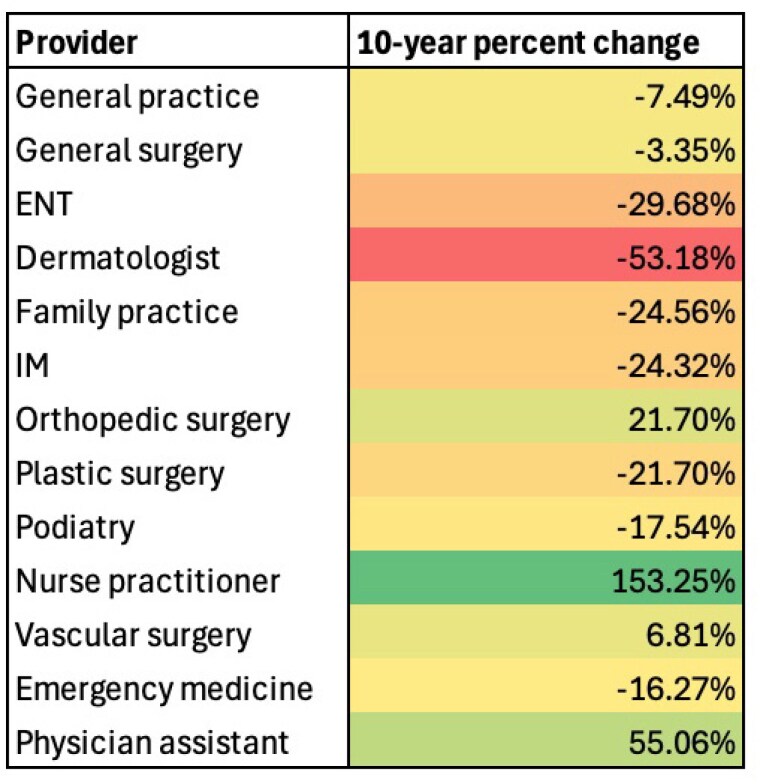# 617 Reimbursement Rates and Characteristics of Burn and Wound Debridement, a Medicare Database Analysis

**DOI:** 10.1093/jbcr/iraf019.246

**Published:** 2025-04-01

**Authors:** Evan Pistone, Thomas Graves, Victoria Cuello, Jeffrey Smith

**Affiliations:** University of Texas Medical Branch; University of Texas Medical Branch; University of Texas Medical Branch; University of Texas Medical Branch

## Abstract

**Introduction:**

Wound debridement is an integral component of treating burns, infections, and chronic wounds. Although there has been increased utilization of non-physician providers and the ability to perform debridement procedures outside of the traditional clinic office visit, it remains unclear whether this has resulted in changes in reimbursement paid out by the Medicare and Medicaid trust fund. This study aims to highlight specific trends in frequency, provider, location, and reimbursement for wound debridement in the Medicare and Medicaid population so that efforts can be made to improve access to care and affordability for historically underserved patients.

**Methods:**

Using data from the Centers for Medicare and Medicaid Services, all claims associated with wound debridement from 2012 to 2022, including debridement for treating burns, were identified through current procedural terminology (CPT) codes. The debridement-related codes included were 11000, 11001, 11045, 11046, and 11047. The burn-related debridement codes included were 16000, 16020, 16025, 16030, 16035, 16036. Over 370,000 Medicare and Medicaid procedures were included, and counts were normalized per 100,000 claims.

**Results:**

Over the study period, the reimbursements for all debridement procedures increased by 120%, far outstripping Consumer Price Index inflation during the same period. However, when stratifying the reimbursement rates by whether the CPT codes are for burn-related debridement, these codes saw a 44% decrease compared to a 152% increase in general wound debridement CPTs. For all debridements, procedures performed by nurse practitioners and physician assistants have increased by 153% and 55%, respectively, while debridements performed by physicians have decreased by between 3% and 53% over the same period. When stratifying reimbursements by CPT codes and providers, a general trend emerges in burn-specific procedures where non-physician providers see increases in their reimbursements while the changes in physician reimbursement are primarily negative.

**Conclusions:**

Reimbursements for burn-specific debridements demonstrate a significant divergence from generalized wound debridement CPT codes, with the gap between the two widening over time. With Reimbursement rates largely down for physician providers performing burn debridement, non-physician providers are receiving larger proportions of medicare reimbursements for most of the selected debridement procedures.

**Applicability of Research to Practice:**

The findings suggest that increased use of non-physician providers for debridement specific for burns has reduced overall reimbursements for these procedures and a shift from physician to non-physician providers. These shifts indicate a need for further study to identify factors driving the divergence between wound and burn debridements to ensure access and affordability are maintained in clinical practice.

**Funding for the Study:**

N/A